# Diaquabis(4-bromo­benzoato-κ*O*)­bis(*N*,*N*′-diethyl­nicotinamide-κ*N* ^1^)zinc(II)

**DOI:** 10.1107/S1600536808027074

**Published:** 2008-08-30

**Authors:** Aslı Öztürk, Tuncer Hökelek, Fureya Elif Özbek, Hacali Necefoğlu

**Affiliations:** a Hacettepe University, Department of Physics, 06800 Beytepe, Ankara, Turkey; b Kafkas University, Department of Chemistry, 63100 Kars, Turkey

## Abstract

The title compound, [Zn(C_7_H_4_BrO_2_)_2_(C_10_H_14_N_2_O)_2_(H_2_O)_2_], is a monomeric complex with the Zn^II^ atom lying on an inversion center. It contains two 4-bromo­benzoate, two diethyl­nicotinamide ligands and two water mol­ecules, all of which are monodentate. The four O atoms in the equatorial plane around the Zn atom form a slightly distorted square-planar arrangement, while the distorted octa­hedral geometry is completed by two N atoms in the axial positions. The methyl group of one of the ethyl groups is disordered over two positions, with occupancies of *ca* 0.65 and 0.35. The two aromatic rings are oriented at an angle of 77.22 (14)°. In the crystal structure, O—H⋯O hydrogen bonds link the mol­ecules into chains along the *a* axis.

## Related literature

For general background, see: Antolini *et al.* (1982 ▶); Nadzhafov *et al.* (1981 ▶). For related literature, see: Clegg *et al.* (1986*a* ▶,*b* ▶); Capilla & Aranda (1979 ▶); Usubaliev *et al.* (1992 ▶); Hökelek *et al.* (1995 ▶, 1997 ▶, 2007 ▶); Hökelek & Necefoğlu (1996 ▶, 1997 ▶); Necefoğlu *et al.* (2002 ▶).
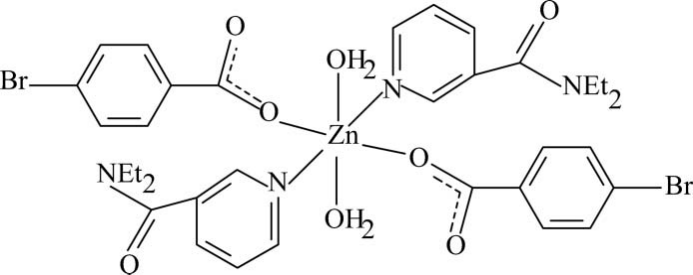


## Experimental

### 

#### Crystal data

[Zn(C_7_H_4_BrO_2_)_2_(C_10_H_14_N_2_O)_2_(H_2_O)_2_]
*M* *_r_* = 857.89
Triclinic, 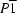

*a* = 7.3761 (14) Å
*b* = 8.677 (2) Å
*c* = 16.072 (3) Å
α = 84.32 (2)°
β = 78.917 (17)°
γ = 67.029 (18)°
*V* = 929.1 (4) Å^3^
*Z* = 1
Mo *K*α radiation
μ = 2.87 mm^−1^
*T* = 294 (2) K
0.40 × 0.25 × 0.15 mm

#### Data collection

Enraf–Nonius TurboCAD-4 diffractometer
Absorption correction: ψ scan (North *et al.*, 1968 ▶) *T*_min_ = 0.467, *T*_max_ = 0.650
4005 measured reflections
3746 independent reflections
2570 reflections with *I* > 2σ(*I*)
*R*_int_ = 0.057
3 standard reflections frequency: 120 min intensity decay: 1%

#### Refinement

*R*[*F* ^2^ > 2σ(*F* ^2^)] = 0.058
*wR*(*F* ^2^) = 0.177
*S* = 1.06
3746 reflections
242 parameters
16 restraints
H atoms treated by a mixture of independent and constrained refinement
Δρ_max_ = 0.87 e Å^−3^
Δρ_min_ = −0.71 e Å^−3^

### 

Data collection: *CAD-4 EXPRESS* (Enraf–Nonius, 1994 ▶); cell refinement: *CAD-4 EXPRESS*; data reduction: *XCAD4* (Harms & Wocadlo, 1995 ▶); program(s) used to solve structure: *SHELXS97* (Sheldrick, 2008 ▶); program(s) used to refine structure: *SHELXL97* (Sheldrick, 2008 ▶); molecular graphics: *ORTEP-3* (Farrugia, 1997 ▶); software used to prepare material for publication: *WinGX* (Farrugia, 1999 ▶).

## Supplementary Material

Crystal structure: contains datablocks I, global. DOI: 10.1107/S1600536808027074/ci2660sup1.cif

Structure factors: contains datablocks I. DOI: 10.1107/S1600536808027074/ci2660Isup2.hkl

Additional supplementary materials:  crystallographic information; 3D view; checkCIF report

## Figures and Tables

**Table:** 

Zn1—O1	2.097 (3)
Zn1—O4	2.143 (3)
Zn1—N1	2.157 (3)

**Table:** 

O1—Zn1—O4^i^	87.83 (12)
O1—Zn1—O4	92.17 (12)
O1—Zn1—N1^i^	88.24 (12)
O4—Zn1—N1^i^	93.29 (13)
O1—Zn1—N1	91.76 (12)
O4—Zn1—N1	86.71 (13)

Symmetry code: (i) 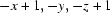.

**Table 2:** Hydrogen-bond geometry (Å, °)

*D*—H⋯*A*	*D*—H	H⋯*A*	*D*⋯*A*	*D*—H⋯*A*
O4—H41⋯O2	0.84 (4)	1.83 (5)	2.658 (5)	168 (3)
O4—H42⋯O3^ii^	0.84 (3)	1.95 (3)	2.786 (6)	169 (2)

Symmetry code: (ii) 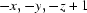.

**Fig. 1.**
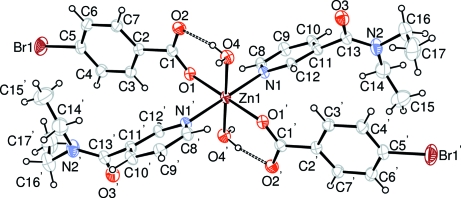
The molecular structure of the title compound with the atom-numbering scheme. Displacement ellipsoids are drawn at the 30% probability level. Hydrogen bonds are shown as dashed lines. Primed atoms are generated by the symmetry operator (1 -x, -y, 1 -z). Only the major disorder component is shown.

**Fig. 2.**
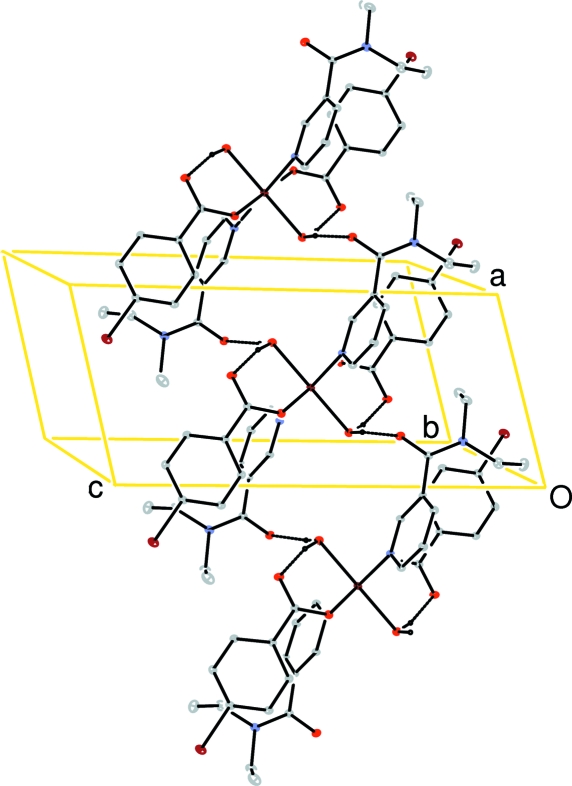
A partial packing diagram of the title compound, viewed down the b axis, showing hydrogen bonds (dashed lines) linking the molecules into chains. H atoms not involved in hydrogen bonding are omitted. The disordered atoms are omitted for clarity. Only the major disorder component is shown.

## supplementary crystallographic information

### Comment

Transition metal complexes with biochemical molecules show interesting physical and/or chemical properties, through which they may find applications in biological systems (Antolini *et al.*, 1982). The structure-function-coordination relationships of the arylcarboxylate ions in Zn^II^ complexes of benzoic acid derivatives may be changed, depending on the nature and position of the substituted groups on the benzene ring, the nature of the additional ligand molecule or solvent, and the medium of the synthesis (Nadzhafov *et al.*, 1981).

The solid-state structures of anhydrous zinc(II) carboxylates include one-dimensional (Clegg *et al.*, 1986*a*), two-dimensional (Clegg *et al.*, 1986*b*) and three-dimensional (Capilla & Aranda, 1979) polymeric motifs of different types, while discrete monomeric complexes with octahedral or tetrahedral coordination geometry are found if water or other donor molecules are coordinated to Zn (Usubaliev *et al.*, 1992).

*N*,*N*-Diethylnicotinamide (DENA) is an important respiratory stimulant. The structures of several complexes obtained by reacting divalent transition metal ions with DENA have been determined, including those of Cu_2_(DENA)_2_(C_6_H_5_COO)_4_ (Hökelek *et al.*, 1995), [Zn_2_(DENA)_2_(C_7_H_5_O_3_)_4_].2H_2_O (Hökelek & Necefoğlu, 1996), [Co(DENA)_2_(C_7_H_5_O_3_)_2_(H_2_O)_2_] (Hökelek & Necefoğlu, 1997) and [Cu(DENA)_2_(C_7_H_4_NO_4_)_2_(H_2_O)_2_] (Hökelek *et al.*, 1997).

The structure determination of the title compound, a zinc complex with two bromobenzoate (BB), two diethylnicotinamide (DENA) ligands and two water molecules, was undertaken in order to determine the properties of the BB and DENA ligands and also to compare the results obtained with those reported previously.

The title compound is a monomeric complex, with the Zn atom on a centre of symmetry. It contains two BB, two DENA ligands and two water molecules (Fig. 1). All ligands are monodentate. The four O atoms (O1, O4, and their symmetry-related atoms, O1', O4') in the equatorial plane around the Zn atom form a slightly distorted square-planar arrangement, while the slightly distorted octahedral coordination geometry is completed by the two N atoms of the DENA ligands (N1, N1') in the axial positions (Table 1 and Fig. 1).

The near equality of the C1—O1 [1.257 (5) Å] and C1—O2 [1.246 (5) Å] bonds in the carboxylate group indicates a delocalized bonding arrangement, rather than localized single and double bonds, as in other zinc(II) complexes: bis(4-hydroxybenzoato-κ*O*)bis(nicotinamide-κ*N*)zinc(II) (Necefoğlu *et al.*, 2002) and diaquabis(*N*,*N*'-diethylnicotinamide-κ*N*)bis(4-fluorobenzoato-κ*O*)- zinc(II) (Hökelek *et al.*, 2007). This may be due to the intramolecular O—H···O hydrogen bonding of the carboxylate O atoms (Table 2). The Zn atom is displaced out of the least-squares plane of the carboxylate group (O1/C1/O2) by 0.885 (1) Å. The planar carboxylate group form dihedral angles of 3.09 (35)° and 80.21 (35)°, respectively, with the benzene (C2-C7) and pyridine (N1/C8-C12) rings. The dihedral angle between C2-C7 and N1/C8-C12 rings is 77.22 (14)°.

As can be seen from the packing diagram (Fig. 2), the molecules are linked into chains, along the *a* axis, by intermolecular O—H···O hydrogen bonds (Table 2).

### Experimental

The title compound was prepared by the reaction of ZnNO_3_ (1.27 g, 10 mmol) in H_2_O (25 ml) and DENA (3.56 g, 20 mmol) in H_2_O (25 ml) with sodium *p*-bromobenzoate (4.46 g, 20 mmol) in H_2_O (100 ml). The mixture was filtered and set aside to crystallize at ambient temperature for several days, giving colourless single crystals.

### Refinement

The H atoms of C14 atom and the C15 methyl group were disordered. During the refinement process the disordered atoms were refined over two positions with occupancies of 0.65 (3) (for C15, H15A, H15B, H15C, H14A and H14B) and 0.35 (3) (for C15A, H15D, H15E, H15F, H14C and H14D). H atoms of water molecule were located in a difference map and refined isotropically with the O-H and H···H distances restrained to 0.84 (1) and 1.37 (2) Å, respectively. The remaining H atoms were positioned geometrically [C-H = 0.93 (aromatic), 0.97 (methylene) and 0.96 Å (methyl)] and constrained to ride on their parent atoms, with *U*_iso_(H) = *xU*_eq_(C), where *x* = 1.5 for methyl H and *x* = 1.2 for all other H atoms.

### Crystal data

**Table:**

[Zn(C_7_H_4_BrO_2_)_2_(C_10_H_14_N_2_O)_2_(H_2_O)_2_]	*Z* = 1
*M**_r_* = 857.89	*F*_000_ = 436
Triclinic, *P*1	*D*_x_ = 1.533 Mg m^−^^3^
Hall symbol: -P 1	Mo *K*α radiation λ = 0.71073 Å
*a* = 7.3761 (14) Å	Cell parameters from 25 reflections
*b* = 8.677 (2) Å	θ = 5.5–13.7º
*c* = 16.072 (3) Å	µ = 2.87 mm^−^^1^
α = 84.32 (2)º	*T* = 294 (2) K
β = 78.917 (17)º	Block, colourless
γ = 67.029 (18)º	0.40 × 0.25 × 0.15 mm
*V* = 929.1 (4) Å^3^	

### Data collection

**Table:**

Enraf–Nonius TurboCAD-4 diffractometer	*R*_int_ = 0.057
Radiation source: fine-focus sealed tube	θ_max_ = 26.3º
Monochromator: graphite	θ_min_ = 2.6º
*T* = 294(2) K	*h* = −8→9
non–profiled ω scans	*k* = 0→10
Absorption correction: ψ scan(North *et al.*, 1968)	*l* = −19→20
*T*_min_ = 0.467, *T*_max_ = 0.650	3 standard reflections
4005 measured reflections	every 120 min
3746 independent reflections	intensity decay: 1%
2570 reflections with *I* > 2σ(*I*)	

### Refinement

**Table:**

Refinement on *F*^2^	Secondary atom site location: difference Fourier map
Least-squares matrix: full	Hydrogen site location: inferred from neighbouring sites
*R*[*F*^2^ > 2σ(*F*^2^)] = 0.058	H atoms treated by a mixture of independent and constrained refinement
*wR*(*F*^2^) = 0.177	*w* = 1/[σ^2^(*F*_o_^2^) + (0.1119*P*)^2^] where *P* = (*F*_o_^2^ + 2*F*_c_^2^)/3
*S* = 1.06	(Δ/σ)_max_ = 0.001
3746 reflections	Δρ_max_ = 0.87 e Å^−^^3^
242 parameters	Δρ_min_ = −0.71 e Å^−^^3^
16 restraints	Extinction correction: none
Primary atom site location: structure-invariant direct methods	

### Special details

**Table:**

Geometry. All e.s.d.'s (except the e.s.d. in the dihedral angle between two l.s. planes) are estimated using the full covariance matrix. The cell e.s.d.'s are taken into account individually in the estimation of e.s.d.'s in distances, angles and torsion angles; correlations between e.s.d.'s in cell parameters are only used when they are defined by crystal symmetry. An approximate (isotropic) treatment of cell e.s.d.'s is used for estimating e.s.d.'s involving l.s. planes.
Refinement. Refinement of *F*^2^ against ALL reflections. The weighted *R*-factor *wR* and goodness of fit *S* are based on *F*^2^, conventional *R*-factors *R* are based on *F*, with *F* set to zero for negative *F*^2^. The threshold expression of *F*^2^ > σ(*F*^2^) is used only for calculating *R*-factors(gt) *etc*. and is not relevant to the choice of reflections for refinement. *R*-factors based on *F*^2^ are statistically about twice as large as those based on *F*, and *R*- factors based on ALL data will be even larger.

### Fractional atomic coordinates and isotropic or equivalent isotropic displacement parameters (Å^2^)

**Table:**

	*x*	*y*	*z*	*U*_iso_*/*U*_eq_	Occ. (<1)
Br1	1.23888 (10)	0.16715 (11)	0.03176 (4)	0.0880 (3)	
Zn1	0.5000	0.0000	0.5000	0.0356 (2)	
O1	0.6060 (4)	0.1244 (4)	0.39536 (19)	0.0414 (7)	
O2	0.4176 (5)	0.1310 (4)	0.3013 (2)	0.0524 (8)	
O3	−0.3317 (5)	0.3246 (4)	0.6209 (2)	0.0541 (9)	
O4	0.2761 (5)	−0.0147 (4)	0.4372 (2)	0.0461 (8)	
H41	0.305 (9)	0.035 (5)	0.3919 (18)	0.08 (2)*	
H42	0.302 (7)	−0.114 (2)	0.425 (3)	0.044 (13)*	
N1	0.2774 (5)	0.2327 (4)	0.5504 (2)	0.0364 (8)	
N2	−0.3276 (7)	0.4115 (6)	0.7461 (3)	0.0619 (12)	
C1	0.5726 (6)	0.1316 (5)	0.3210 (3)	0.0392 (10)	
C2	0.7355 (6)	0.1412 (5)	0.2502 (3)	0.0373 (9)	
C3	0.9095 (7)	0.1478 (6)	0.2676 (3)	0.0420 (10)	
H3	0.9259	0.1455	0.3237	0.050*	
C4	1.0587 (7)	0.1578 (6)	0.2035 (3)	0.0483 (11)	
H4	1.1739	0.1640	0.2157	0.058*	
C5	1.0324 (7)	0.1583 (6)	0.1206 (3)	0.0503 (12)	
C6	0.8638 (8)	0.1483 (7)	0.1016 (3)	0.0536 (12)	
H6	0.8493	0.1478	0.0454	0.064*	
C7	0.7161 (7)	0.1390 (6)	0.1666 (3)	0.0446 (11)	
H7	0.6020	0.1313	0.1540	0.054*	
C8	0.3051 (6)	0.3789 (5)	0.5402 (3)	0.0416 (10)	
H8	0.4248	0.3804	0.5093	0.050*	
C9	0.1628 (7)	0.5253 (6)	0.5737 (3)	0.0465 (11)	
H9	0.1870	0.6237	0.5659	0.056*	
C10	−0.0151 (7)	0.5256 (6)	0.6188 (3)	0.0451 (11)	
H10	−0.1127	0.6240	0.6419	0.054*	
C11	−0.0477 (6)	0.3765 (5)	0.6296 (3)	0.0376 (9)	
C12	0.1022 (6)	0.2355 (5)	0.5923 (3)	0.0371 (9)	
H12	0.0790	0.1369	0.5967	0.045*	
C13	−0.2459 (7)	0.3684 (6)	0.6665 (3)	0.0441 (11)	
C14	−0.2330 (11)	0.4635 (9)	0.8056 (4)	0.0805 (18)	
H14A	−0.1040	0.4607	0.7761	0.097*	0.65 (3)
H14B	−0.3146	0.5788	0.8202	0.097*	0.65 (3)
H14C	−0.3291	0.5430	0.8456	0.097*	0.35 (3)
H14D	−0.1387	0.5090	0.7755	0.097*	0.35 (3)
C15	−0.203 (3)	0.365 (3)	0.8832 (11)	0.109 (6)	0.65 (3)
H15A	−0.1407	0.4092	0.9167	0.164*	0.65 (3)
H15B	−0.1197	0.2511	0.8701	0.164*	0.65 (3)
H15C	−0.3302	0.3704	0.9145	0.164*	0.65 (3)
C15A	−0.128 (4)	0.301 (2)	0.837 (3)	0.099 (10)	0.35 (3)
H15D	−0.0473	0.3079	0.8755	0.148*	0.35 (3)
H15E	−0.0432	0.2327	0.7901	0.148*	0.35 (3)
H15F	−0.2208	0.2534	0.8652	0.148*	0.35 (3)
C16	−0.5345 (9)	0.4168 (8)	0.7762 (5)	0.0759 (18)	
H16A	−0.5968	0.4889	0.8246	0.091*	
H16B	−0.6138	0.4624	0.7315	0.091*	
C17	−0.5281 (12)	0.2459 (9)	0.8008 (5)	0.103 (3)	
H17A	−0.4543	0.2029	0.8467	0.154*	
H17B	−0.4641	0.1743	0.7532	0.154*	
H17C	−0.6617	0.2498	0.8184	0.154*	

### Atomic displacement parameters (Å^2^)

**Table:**

	*U*^11^	*U*^22^	*U*^33^	*U*^12^	*U*^13^	*U*^23^
Br1	0.0663 (5)	0.1420 (7)	0.0592 (4)	−0.0549 (5)	0.0153 (3)	−0.0050 (4)
Zn1	0.0288 (4)	0.0375 (4)	0.0394 (4)	−0.0128 (3)	0.0004 (3)	−0.0065 (3)
O1	0.0395 (17)	0.0430 (17)	0.0413 (18)	−0.0169 (14)	−0.0014 (13)	−0.0052 (13)
O2	0.0335 (17)	0.070 (2)	0.055 (2)	−0.0237 (16)	−0.0073 (14)	0.0055 (17)
O3	0.0423 (19)	0.068 (2)	0.062 (2)	−0.0294 (17)	−0.0069 (16)	−0.0144 (17)
O4	0.0414 (18)	0.048 (2)	0.055 (2)	−0.0232 (16)	−0.0061 (15)	−0.0058 (16)
N1	0.0312 (18)	0.0340 (19)	0.042 (2)	−0.0118 (15)	−0.0010 (14)	−0.0038 (15)
N2	0.050 (3)	0.082 (3)	0.059 (3)	−0.036 (2)	0.008 (2)	−0.016 (2)
C1	0.036 (2)	0.030 (2)	0.048 (3)	−0.0112 (18)	−0.0031 (19)	−0.0024 (18)
C2	0.038 (2)	0.033 (2)	0.041 (2)	−0.0152 (18)	−0.0018 (18)	−0.0043 (17)
C3	0.040 (2)	0.047 (3)	0.041 (2)	−0.018 (2)	−0.0066 (19)	−0.0036 (19)
C4	0.039 (3)	0.058 (3)	0.051 (3)	−0.024 (2)	−0.003 (2)	−0.001 (2)
C5	0.041 (3)	0.057 (3)	0.049 (3)	−0.020 (2)	0.005 (2)	−0.005 (2)
C6	0.049 (3)	0.073 (3)	0.038 (3)	−0.022 (3)	−0.004 (2)	−0.002 (2)
C7	0.037 (2)	0.056 (3)	0.043 (3)	−0.020 (2)	−0.0056 (19)	−0.003 (2)
C8	0.033 (2)	0.047 (3)	0.048 (3)	−0.020 (2)	−0.0011 (19)	−0.005 (2)
C9	0.047 (3)	0.036 (2)	0.058 (3)	−0.018 (2)	−0.004 (2)	−0.008 (2)
C10	0.036 (2)	0.038 (2)	0.057 (3)	−0.0086 (19)	−0.004 (2)	−0.014 (2)
C11	0.031 (2)	0.041 (2)	0.040 (2)	−0.0115 (17)	−0.0050 (17)	−0.0080 (18)
C12	0.030 (2)	0.038 (2)	0.045 (2)	−0.0152 (18)	−0.0011 (17)	−0.0041 (18)
C13	0.036 (2)	0.043 (2)	0.052 (3)	−0.014 (2)	−0.002 (2)	−0.009 (2)
C14	0.080 (5)	0.080 (4)	0.075 (4)	−0.028 (4)	−0.003 (3)	−0.006 (3)
C15	0.118 (9)	0.139 (10)	0.077 (8)	−0.048 (7)	−0.038 (7)	0.006 (7)
C15A	0.092 (12)	0.089 (12)	0.118 (14)	−0.038 (9)	−0.009 (9)	−0.013 (8)
C16	0.059 (4)	0.067 (4)	0.094 (5)	−0.025 (3)	0.015 (3)	−0.020 (3)
C17	0.116 (6)	0.079 (5)	0.102 (6)	−0.047 (5)	0.025 (5)	0.001 (4)

### Geometric parameters (Å, °)

**Table:**

Br1—C5	1.897 (5)	C8—C9	1.372 (6)
Zn1—O1^i^	2.097 (3)	C8—H8	0.93
Zn1—O1	2.097 (3)	C9—C10	1.371 (6)
Zn1—O4^i^	2.143 (3)	C9—H9	0.93
Zn1—O4	2.143 (3)	C10—C11	1.394 (6)
Zn1—N1^i^	2.157 (3)	C10—H10	0.93
Zn1—N1	2.157 (3)	C11—C12	1.383 (6)
O1—C1	1.257 (5)	C11—C13	1.493 (6)
O2—C1	1.246 (5)	C12—H12	0.93
O3—C13	1.226 (6)	C14—C15A	1.409 (16)
O4—H41	0.84 (4)	C14—C15	1.441 (12)
O4—H42	0.84 (3)	C14—H14A	0.97
N1—C12	1.330 (5)	C14—H14B	0.97
N1—C8	1.352 (5)	C14—H14C	0.96
N2—C13	1.328 (6)	C14—H14D	0.96
N2—C14	1.481 (8)	C15—H15A	0.96
N2—C16	1.494 (7)	C15—H15B	0.96
C1—C2	1.510 (6)	C15—H15C	0.96
C2—C7	1.381 (6)	C15A—H15D	0.96
C2—C3	1.389 (6)	C15A—H15E	0.96
C3—C4	1.379 (6)	C15A—H15F	0.96
C3—H3	0.93	C16—C17	1.481 (9)
C4—C5	1.382 (7)	C16—H16A	0.97
C4—H4	0.93	C16—H16B	0.97
C5—C6	1.373 (7)	C17—H17A	0.96
C6—C7	1.378 (6)	C17—H17B	0.96
C6—H6	0.93	C17—H17C	0.96
C7—H7	0.93		
			
O1^i^—Zn1—O1	180	C9—C10—C11	119.1 (4)
O1^i^—Zn1—O4^i^	92.17 (12)	C9—C10—H10	120.4
O1—Zn1—O4^i^	87.83 (12)	C11—C10—H10	120.4
O1^i^—Zn1—O4	87.83 (12)	C12—C11—C10	117.5 (4)
O1—Zn1—O4	92.17 (12)	C12—C11—C13	118.7 (4)
O4^i^—Zn1—O4	180	C10—C11—C13	123.1 (4)
O1^i^—Zn1—N1^i^	91.76 (12)	N1—C12—C11	123.9 (4)
O1—Zn1—N1^i^	88.24 (12)	N1—C12—H12	118.0
O4^i^—Zn1—N1^i^	86.71 (13)	C11—C12—H12	118.0
O4—Zn1—N1^i^	93.29 (13)	O3—C13—N2	121.3 (4)
O1^i^—Zn1—N1	88.24 (12)	O3—C13—C11	118.3 (4)
O1—Zn1—N1	91.76 (12)	N2—C13—C11	120.3 (4)
O4^i^—Zn1—N1	93.29 (13)	C15A—C14—N2	96.5 (14)
O4—Zn1—N1	86.71 (13)	C15—C14—N2	116.4 (8)
N1^i^—Zn1—N1	180	C15—C14—H14A	108.2
C1—O1—Zn1	126.3 (3)	N2—C14—H14A	108.2
Zn1—O4—H41	96 (4)	C15—C14—H14B	108.2
Zn1—O4—H42	111 (3)	N2—C14—H14B	108.2
H41—O4—H42	107 (2)	H14A—C14—H14B	107.3
C12—N1—C8	117.5 (3)	C15A—C14—H14C	117.8
C12—N1—Zn1	119.3 (3)	N2—C14—H14C	112.5
C8—N1—Zn1	123.1 (3)	C15A—C14—H14D	108.9
C13—N2—C14	124.7 (5)	N2—C14—H14D	111.0
C13—N2—C16	117.5 (5)	H14C—C14—H14D	109.6
C14—N2—C16	117.7 (5)	C14—C15—H15A	109.5
O2—C1—O1	125.5 (4)	H14C—C15—H15A	94.4
O2—C1—C2	117.9 (4)	C14—C15—H15B	109.5
O1—C1—C2	116.7 (4)	H14C—C15—H15B	145.2
C7—C2—C3	118.7 (4)	H15A—C15—H15B	109.5
C7—C2—C1	120.3 (4)	C14—C15—H15C	109.5
C3—C2—C1	120.9 (4)	H14C—C15—H15C	84.6
C4—C3—C2	121.4 (5)	H15A—C15—H15C	109.5
C4—C3—H3	119.3	H15B—C15—H15C	109.5
C2—C3—H3	119.3	C14—C15A—H15D	109.5
C3—C4—C5	118.2 (5)	C14—C15A—H15E	109.5
C3—C4—H4	120.9	H15D—C15A—H15E	109.5
C5—C4—H4	120.9	C14—C15A—H15F	109.5
C6—C5—C4	121.6 (4)	H15D—C15A—H15F	109.5
C6—C5—Br1	119.7 (4)	H15E—C15A—H15F	109.5
C4—C5—Br1	118.6 (4)	C17—C16—N2	110.0 (6)
C5—C6—C7	119.3 (5)	C17—C16—H16A	109.7
C5—C6—H6	120.3	N2—C16—H16A	109.7
C7—C6—H6	120.3	C17—C16—H16B	109.7
C6—C7—C2	120.7 (4)	N2—C16—H16B	109.7
C6—C7—H7	119.6	H16A—C16—H16B	108.2
C2—C7—H7	119.6	C16—C17—H17A	109.5
N1—C8—C9	122.3 (4)	C16—C17—H17B	109.5
N1—C8—H8	118.8	H17A—C17—H17B	109.5
C9—C8—H8	118.8	C16—C17—H17C	109.5
C8—C9—C10	119.6 (4)	H17A—C17—H17C	109.5
C8—C9—H9	120.2	H17B—C17—H17C	109.5
C10—C9—H9	120.2		
			
O4^i^—Zn1—O1—C1	163.0 (3)	C3—C2—C7—C6	−1.9 (7)
O4—Zn1—O1—C1	−17.0 (3)	C1—C2—C7—C6	179.8 (4)
N1^i^—Zn1—O1—C1	76.3 (3)	C12—N1—C8—C9	2.3 (7)
N1—Zn1—O1—C1	−103.7 (3)	Zn1—N1—C8—C9	−179.1 (3)
O1^i^—Zn1—N1—C12	−33.6 (3)	N1—C8—C9—C10	−0.5 (7)
O1—Zn1—N1—C12	146.4 (3)	C8—C9—C10—C11	0.0 (7)
O4^i^—Zn1—N1—C12	−125.7 (3)	C9—C10—C11—C12	−1.2 (7)
O4—Zn1—N1—C12	54.3 (3)	C9—C10—C11—C13	−170.8 (5)
O1^i^—Zn1—N1—C8	147.8 (3)	C8—N1—C12—C11	−3.7 (6)
O1—Zn1—N1—C8	−32.2 (3)	Zn1—N1—C12—C11	177.6 (3)
O4^i^—Zn1—N1—C8	55.7 (4)	C10—C11—C12—N1	3.2 (7)
O4—Zn1—N1—C8	−124.3 (4)	C13—C11—C12—N1	173.3 (4)
Zn1—O1—C1—O2	31.6 (6)	C14—N2—C13—O3	179.1 (5)
Zn1—O1—C1—C2	−148.2 (3)	C16—N2—C13—O3	−4.4 (7)
O2—C1—C2—C7	−3.8 (6)	C14—N2—C13—C11	−2.4 (8)
O1—C1—C2—C7	176.0 (4)	C16—N2—C13—C11	174.1 (5)
O2—C1—C2—C3	177.9 (4)	C12—C11—C13—O3	−54.7 (6)
O1—C1—C2—C3	−2.3 (6)	C10—C11—C13—O3	114.9 (5)
C7—C2—C3—C4	2.2 (6)	C12—C11—C13—N2	126.8 (5)
C1—C2—C3—C4	−179.5 (4)	C10—C11—C13—N2	−63.7 (7)
C2—C3—C4—C5	−1.0 (7)	C13—N2—C14—C15A	−87.7 (16)
C3—C4—C5—C6	−0.4 (8)	C16—N2—C14—C15A	95.7 (16)
C3—C4—C5—Br1	−178.7 (4)	C13—N2—C14—C15	−121.7 (12)
C4—C5—C6—C7	0.6 (8)	C16—N2—C14—C15	61.7 (13)
Br1—C5—C6—C7	178.9 (4)	C13—N2—C16—C17	81.6 (7)
C5—C6—C7—C2	0.6 (8)	C14—N2—C16—C17	−101.6 (7)

Symmetry codes: (i) −*x*+1, −*y*, −*z*+1.

### Hydrogen-bond geometry (Å, °)

**Table:**

*D*—H···*A*	*D*—H	H···*A*	*D*···*A*	*D*—H···*A*
O4—H41···O2	0.84 (4)	1.83 (5)	2.658 (5)	168 (3)
O4—H42···O3^ii^	0.84 (3)	1.95 (3)	2.786 (6)	169 (2)

Symmetry codes: (ii) −*x*, −*y*, −*z*+1.
